# New York State Board of Veterinary Medical Examiners

**Published:** 1901-11

**Authors:** 


### NEW YORK STATE BOARD OF VETERINARY MEDICAL EXAMINERS.

Dr. William H. Kelly, Secretary of the Board, reports the following changes in the law: "Chapter 644. Five hundred dollars, or as much thereof as may be available for proper expenses incurred in the administration of the veterinary law, and the apportionment of the surplus among the veterinary medical examiners, as therein provided; also payable from fees received, involving no expense to the State, under Section 49," etc. The second amendment admits to the licensing examination any graduate who matriculated prior to January 1, 1895, and any person who practised prior to 1886. The following candidates were licensed at the examinations of 1900 and 1901: John William Fink, September, 1900; Charles Frederick Flocken, Charles Joseph Jones, Clarence Earl Shaw, Joseph Lot Wilder, Edward A. A. Grange, Raymond Clinton Reed—all of June, 1901.

